# Large-scale column-free purification of bovine F-ATP synthase

**DOI:** 10.1016/j.jbc.2023.105603

**Published:** 2023-12-28

**Authors:** Chimari Jiko, Yukio Morimoto, Tomitake Tsukihara, Christoph Gerle

**Affiliations:** 1 Division of Radiation Life Science, Institute for Integrated Radiation and Nuclear Science, Kyoto University, Osaka, Japan; 2 Department of Life Science, Graduate School of Life Science, University of Hyogo, Koto, Kamigori, Hyogo, Japan; 3 Laboratory for Protein Crystallography, Institute for Protein Research, Osaka University, Osaka, Japan; 4 Life Science Research Infrastructure Group, RIKEN SPring-8 Center, Kouto, Hyogo, Japan

**Keywords:** membrane protein, purification, bioenergetics, mitochondria, rotary ATPase, gradient centrifugation, supercomplex, F-ATP synthase oligomer

## Abstract

Mammalian F-ATP synthase is central to mitochondrial bioenergetics and is present in the inner mitochondrial membrane in a dynamic oligomeric state of higher oligomers, tetramers, dimers, and monomers. *In vitro* investigations of mammalian F-ATP synthase are often limited by the ability to purify the oligomeric forms present *in vivo* at a quantity, stability, and purity that meets the demand of the planned experiment. We developed a purification approach for the isolation of bovine F-ATP synthase from heart muscle mitochondria that uses a combination of buffer conditions favoring inhibitor factor 1 binding and sucrose density gradient ultracentrifugation to yield stable complexes at high purity in the milligram range. By tuning the glyco-diosgenin to lauryl maltose neopentyl glycol ratio in a final gradient, fractions that are either enriched in tetrameric or monomeric F-ATP synthase can be obtained. It is expected that this large-scale column-free purification strategy broadens the spectrum of *in vitro* investigation on mammalian F-ATP synthase.

Mitochondrial F-ATP synthase is an inner membrane bound rotary nanomachine essential for the interconversion of proton motive force and ATP (1, 2)⁠. Unlike its bacterial and chloroplast counterparts, mitochondrial F-ATP synthase is present in diverse, species dependent oligomeric forms and possesses likewise diverse and species dependent sets of supernumerary subunits that are not directly involved in ATP synthesis (3, 4, 5, 6, 7, 8, 9, 10, 11, 12)⁠. In mammals, at times of a proton motive force insufficient for ATP synthesis futile ATP hydrolysis is avoided *via* the binding of the natural inhibitor protein inhibitor factor 1 (IF1) (13, 14, 15, 16), a process that is cell type dependent and under active investigation (17, 18)⁠. Binding of IF1 to the hydrophilic F_1_ domain is thought to stabilize oligomerization (14)⁠, a notion that has been greatly strengthened by the structure of the IF1 bound porcine F-ATP synthase tetramer in which two neighboring dimers are connected through two interlinking IF1 dimers (11)⁠. A recent study on mammalian F-ATP synthase as a druggable complex for the alleviation of mitochondrial disorder highlighted the usefulness of purified F-ATP synthase for *in vitro* studies in drug discovery (19)⁠. Likewise, the newly discovered role of mammalian F-ATP synthase as the molecular identity of the mitochondrial permeability transition pore (mPTP) raises great interest in the ability to conduct conclusive *in vitro* experiments using purified F-ATP synthase complexes in their various oligomeric forms (20, 21, 22)⁠. The above underlines the need to develop novel protocols that ease the purification and experimental handling of mammalian F-ATP synthase in both monomeric and oligomeric states.

Since its discovery as the site of ATP synthesis from ADP and P_i_ more than 50 years ago (23, 24)⁠, mammalian F-ATP synthase isolated from heart muscle tissue mitochondria has been the subject of investigation on the structure and function of mitochondrial F-ATP synthase. Concomitantly, various strategies for the isolation of mitochondrial F-ATP synthase from mammalian cells were developed (25, 26, 27, 28, 29, 30, 31)⁠.

Early studies succeeded to purify its water soluble F_1_ domain to high purity, eventually allowing the growth of well diffracting 3D crystals for structure determination by X-ray crystallography (32, 33)⁠. Isolation of the whole mammalian F-ATP synthase complex, however, proved to be challenging when using conventional detergents, often yielding complexes of poor stability and homogeneity. Recently, the use of very mild, lipid like detergents has dramatically improved the situation with regard to the stability of mammalian F-ATP synthase during isolation of the complex. Namely, the natural compound digitonin, its synthetic stand-in glyco-diosgenin (GDN) (34)⁠ and the two acyl chain tailed lauryl maltose neopentyl glycol (LMNG) (35)⁠ have become the detergents of choice for isolation of intact F-ATP synthase from mammalian sources. All of them greatly alleviate the instability problem and enabled the successful large-scale purification of F-ATP synthase from heart muscle tissue of large animals or human cell culture for structural and functional studies (9, 10, 11, 19, 21, 36, 37, 38, 39, 40)⁠. However, isolation of highly stable monomeric and especially oligomeric mammalian F-ATP synthase at high yield and purity is still challenging, leaving ample of room for improvement in purification procedures. For a discussion of challenges in current *in vitro* investigations of mammalian ATP synthase, refer to the recent review by Bernardi, Gerle, and colleagues on the mPTP (41). Perhaps the most desirable of all oligomeric forms of mammalian ATP synthase is the IF1 bound tetrameric F-ATP synthase, since it represents a minimal oligomeric structural unit which can be expected to be of high physiological relevance. However, enrichment of IF1 bound tetrameric ATP synthase from mammalian source at high yield and in the absence of contaminating respiratory complexes has not been achieved yet (11).

In all of the reported large-scale purifications of mitochondrial F-ATP synthase from mammalian cells, chromatography steps such as anion exchange chromatography, size exclusion chromatography, or IF1 affinity resins are used as necessary tools for the removal of contaminant proteins (9, 10, 11, 21, 37, 42)⁠. This use of chromatography steps limits the yield of oligomeric ATP synthase and can lead to the partial loss of subunits (37). In contrast, it has been shown that for large membrane protein complexes from higher organisms, isolation procedures that exclude any chromatography steps can be beneficial for obtaining well behaving protein suitable for structural and functional studies (43, 44, 45)⁠.

We reasoned that *in vitro* studies of mammalian F-ATP synthase could greatly benefit, if novel strategies for large-scale column-free purification would be developed. This might enable design of purification strategies tailored to the isolation of specific oligomeric states while avoiding any damage during the purification process. Density gradient ultracentrifugation is a powerful method for column-free, mild isolation of large protein complexes widely used in photosynthetic and ribosomal research fields (44, 46)⁠. The size similarity of monomeric, dimeric, and tetrameric F-ATP synthase to other complexes of the mitochondrial respiratory chain, however, complicates application of this approach to mammalian F-ATP synthase. Given that higher oligomers of F-ATP synthase are much larger than respiratory supercomplexes, and that IF1 binding can stabilize oligomer formation, we surmised that a combination of IF1 binding and density gradient ultracentrifugation might be a possible avenue for developing novel procedures for large-scale column-free purification.

Here, we describe the successful large-scale column-free purification of oligomeric and monomeric F-ATP synthase from bovine heart mitochondria at high yield and purity by sucrose density gradient ultracentrifugation.

## Results

**Figure 1:**
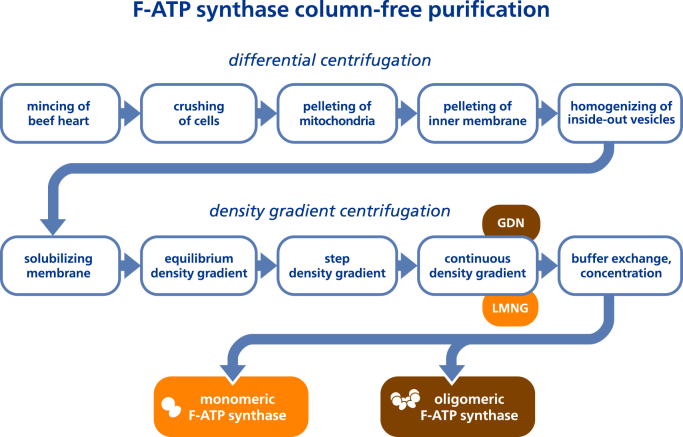
**Flow chart of steps in the large-scale column-free purification of F-ATP synthase from bovine heart muscle tissue mitochondria**. Differential centrifugation is used to obtain large amounts of pure inner mitochondrial membranes. Solubilized membranes are subjected to three sucrose density gradient ultracentrifugation steps which can be tuned *via* the use of either LMNG or GDN to yield oligomeric or monomeric F-ATP synthase. GDN, glyco-diosgenin; LMNG, lauryl maltose neopentyl glycol.

**Figure 2:**
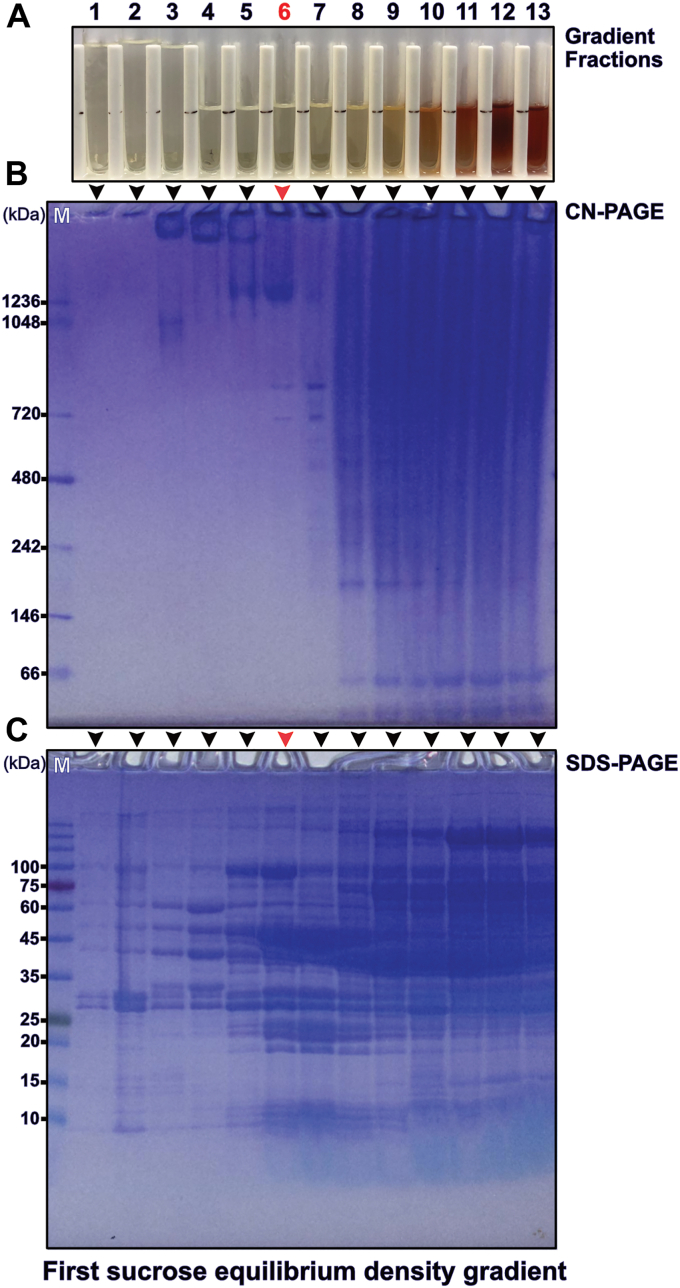
**Fractionation of the equilibrium sucrose density gradient.***A*, fractions from a gradient (Fig. S1*F*, *after*) as collected from bottom to top. The fraction used for further purification is marked by a six in *red font*. *B*, clear native gel electrophoresis of the fractions shown in (*A*) and indicated by *arrow heads*. The *red arrow* head indicates the lane corresponding to fraction six in (*A*) and M indicates the native molecular weight marker lane. *C*, denaturing gel electrophoresis of the fractions as shown in (*A*) and indicated by arrowheads with the *red arrowhead* marking the fraction used for further purification. A molecular weight marker lane is marked by M.

**Figure S1:**
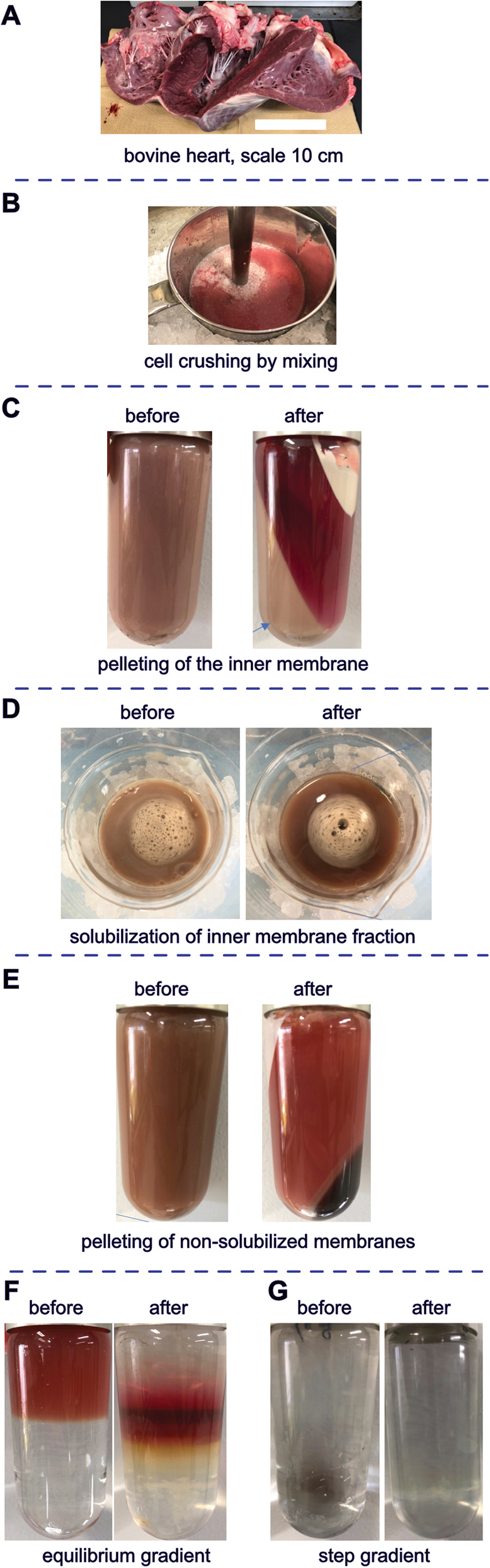


**Figure S2:**
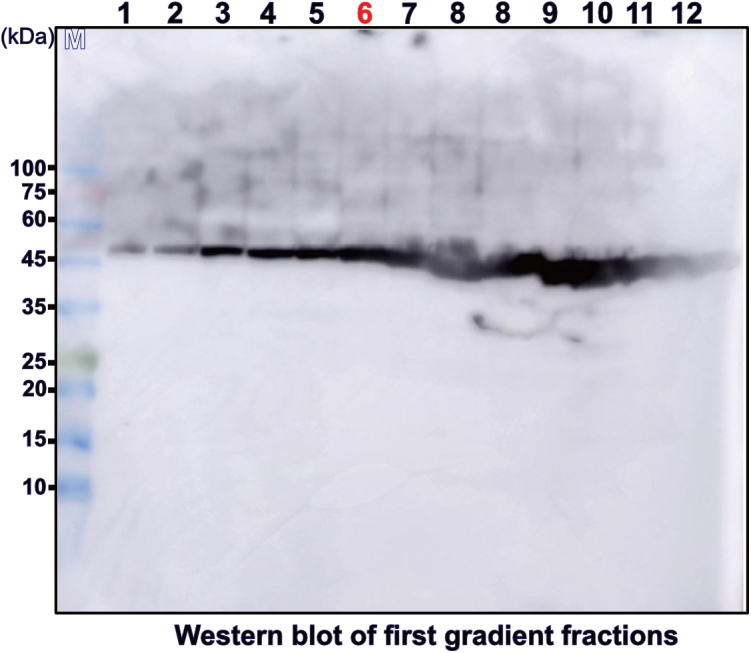


**Figure S3:**
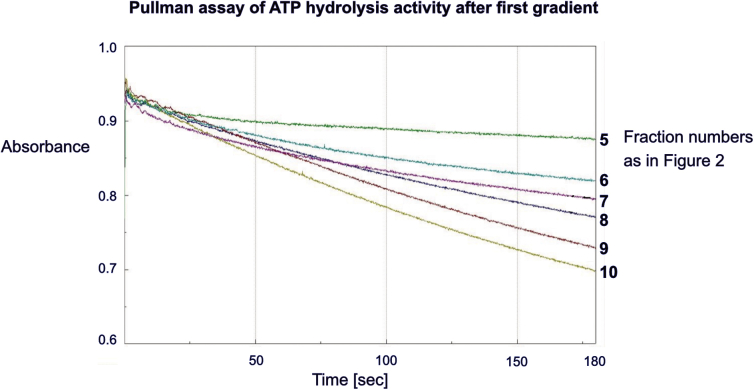


**Figure S4:**
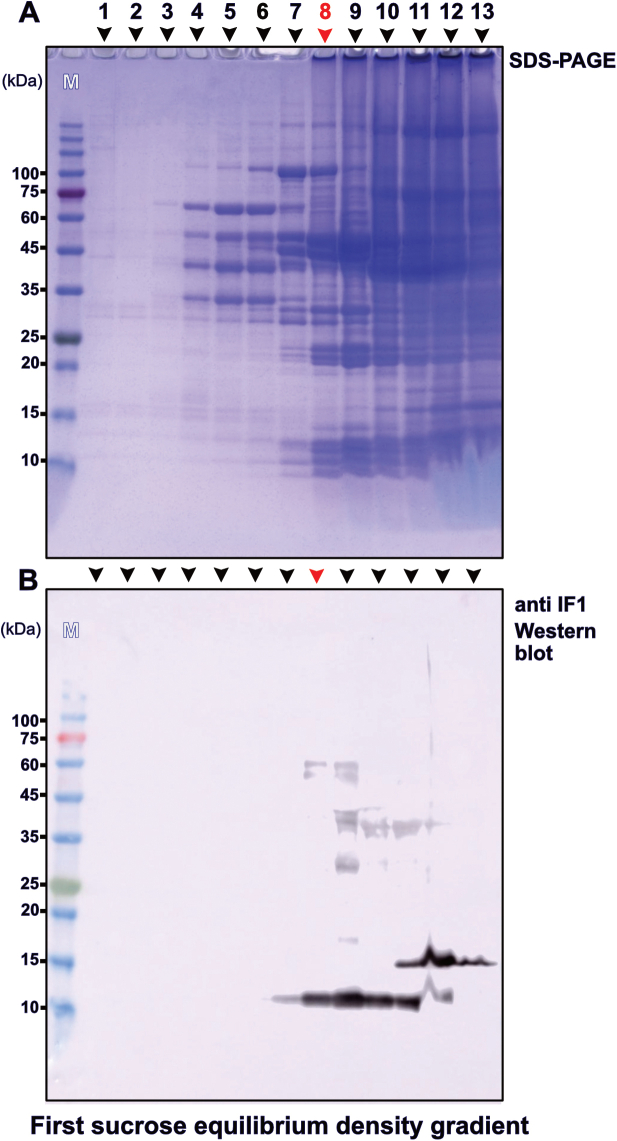


In this study, we established a large-scale column-free purification procedure for bovine heart mitochondrial F-ATP synthase (Fig. 1 for a workflow chart). We demonstrate that this is feasible when taking advantage of the very large size and molecular weight of IF1-stabilized oligomers of heart muscle tissue F-ATP synthase. In the first step of this approach, we made use of well-established procedures to obtain large amounts of highly pure mitochondrial inner membranes from bovine heart muscle tissue (30, 43). This is achieved by differential centrifugation of first mitochondria and then inside-out vesicles of the mitochondrial inner membrane. Experimental conditions for this type of mitochondrial inner membrane isolation had originally been established for the high-resolution structure determination of bovine cytochrome *c* oxidase by X-ray crystallography (47)⁠. Mass spectrometry demonstrated mitochondrial inner membranes prepared in this way are only minimally contaminated with the outer mitochondrial membrane or other cellular membranes (48)⁠. To strengthen the binding of IF1 to mitochondrial F-ATP synthase during isolation of the inner membrane fraction, buffer conditions were modified to a lowered pH and the presence of Mg·ADP (49))⁠. These modifications did not affect the high yield of ∼70 g inner membrane fraction typically obtainable from 600 g of lean heart muscle tissue (Fig. S1, *A*–*C*). A mixture of deoxycholate and decyl maltoside (DM) had been previously established to be efficient for the solubilization of the highly packed mitochondrial inner membranes (30)⁠. However, intending to stabilize larger oligomers of IF1 bound F-ATP synthase, we supplemented this detergent mixture with the additional mixture of GDN and LMNG (Fig. S1, *D* and *E*). Both novel detergents are known for their ability to stabilize fragile membrane complexes during membrane solubilization and purification. In combination with a slightly acidic pH and the presence of Mg·ADP for tight IF1 binding, a stabilization of higher oligomers of F-ATP synthase was expected. Under these conditions it was possible to subject the solubilized inner membranes to a 42 h equilibrium sucrose density gradient centrifugation and obtain almost colorless fractions, indicative of the absence of other iron-containing mitochondrial complexes of the respiratory chain (Figs. 2*A* and S1*F*). When examined by denaturing SDS-PAGE, strong bands distinct for the α and β subunits of the F-ATP synthase could be discerned (Fig. 2*C*). Western blots using an antibody specific for the F-ATP synthase β subunit confirmed the presence of the β subunit in all fractions, indicating the spread of F-ATP synthase over the sucrose gradient (Fig. S2). The widespread presence of F-ATP synthase over basically all fractions is presumably the result of higher oligomers of various sizes. Clear native polyacrylamide gel electrophoresis (CN-PAGE) had been specifically developed to allow native gel electrophoresis of fragile mammalian F-ATP synthase oligomers (50)⁠. Therefore, together with SDS-PAGE we additionally used CN-PAGE to monitor each sucrose density gradient fraction. In the almost colorless fractions bands corresponding to high molecular weight typical for oligomeric F-ATP synthase could be observed (21)⁠. Importantly lower molecular bands stemming from broken complexes and dissociated F_1_ domains were not observed (Fig. 2*B*). When we probed the ATP hydrolysis activity of the F-ATP synthase enriched fractions we detected little ATP hydrolysis activity, again indicative of absence of dissociated F_1_ domains (Fig. S3). The high molecular weight of these ATP hydrolysis “silenced” F-ATP synthase fractions suggest the presence of IF1 stabilized higher oligomers of F-ATP synthase. This expected presence of IF1 in fractions containing a high load of F-ATP synthase was confirmed by Western blotting using an IF1 specific antibody (Fig. S4). In contrast, we could observe higher ATP hydrolase activity in the upper colored fractions of the gradient, which is suggestive of the presence of F-ATP synthase of smaller oligomeric state and a smaller proportion of IF1 binding. As a result, a single equilibrium sucrose density gradient allows, under the experimental conditions used here, the separation of large oligomers of IF1-bound F-ATP synthase in fractions that already contain few other contaminants.

**Figure 3:**
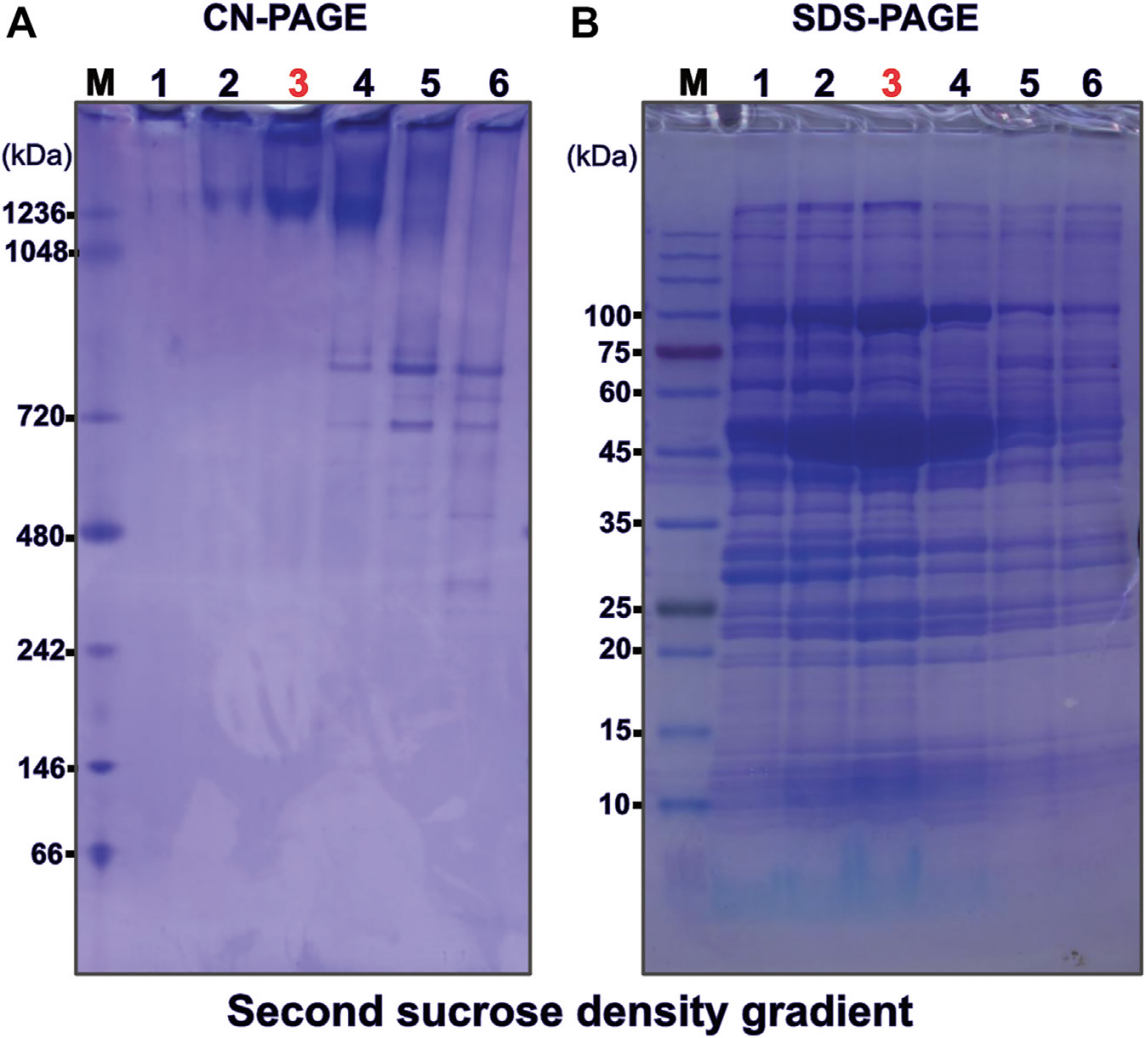
**Native and denaturing gel electrophoresis of the second sucrose step density gradient (**Fig. S1***G*, after)**. *A*, fractions from bottom to top as analyzed by clear native gel electrophoresis with the fraction used for further purification indicated by three in *red font*. The molecular weight marker lane on the far *left* is marked by “M”. *B*, denaturing gel electrophoresis of the same fractions that were used in (*A*) with a three in *red font* indication the fraction used for further purification. The molecular weight marker lane on the far left is marked by “M”.

Next, these colorless, “ATP hydrolase activity silent” F-ATP synthase enriched fractions were pooled and used for a second sucrose step gradient ultracentrifugation for the removal of almost all remaining contaminants. This second sucrose density gradient yielded relatively pure, but oligomeric mixed F-ATP synthase. CN-PAGE of gradient fractions did not show any bands at lower molecular weight indicative of a high stability of the obtained F-ATP synthase fractions also after the second density gradient (Figs. 3 and S1*G*). A typical yield for the indicated target fraction number three (Fig. 3) is about 40 mg of total protein.

**Figure 4:**
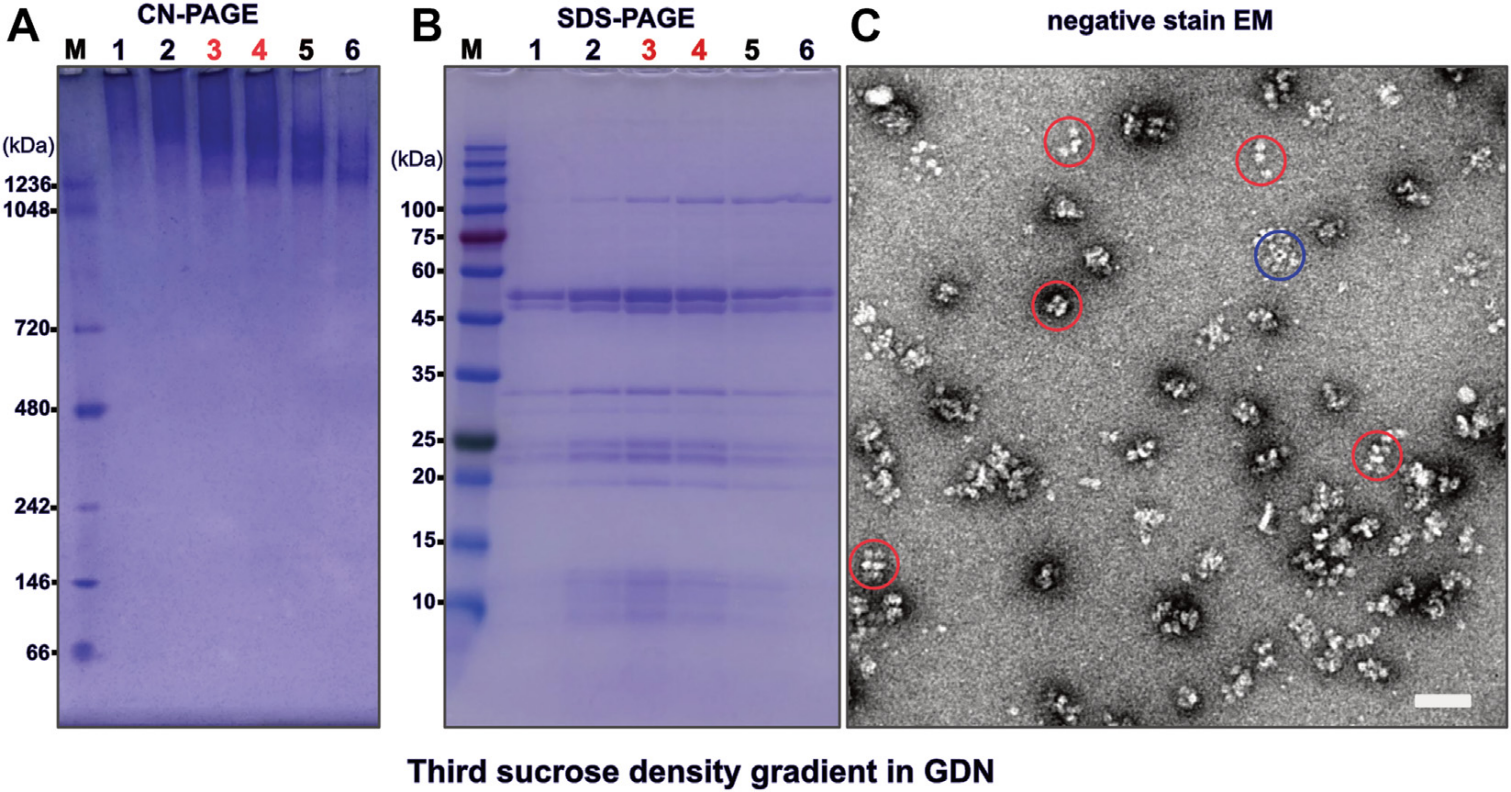
**Analysis of the third continuous sucrose GDN density gradient fractions by native gel electrophoresis, denaturing gel electrophoresis, and negative stain electron microscopy**. *A*, clear native gel electrophoresis of fractions collected from bottom to top of the gradient with the fractions enriched in tetrameric F-ATP synthase and used for negative stain electron microscopy marked in *red font*. The molecular weight marker lane on the far left is marked by “M”. *B*, denaturing gel electrophoresis of fractions collected from bottom to top of the gradient as analyzed in (*A*). The fractions enriched in tetrameric F-ATP synthase and used for negative stain electron microscopy are marked in *red font*. The molecular weight marker lane on the far left is marked by “M”. *C*, negative stain electron microscopy of fraction 4. Some of the F-ATP synthase complexes that appear to be tetrameric F-ATP synthase are encircled in *red*. A putative contaminant 2-oxoglutarate dehydrogenase complex is encircled in *blue*. The scale bar represents 50 nm. GDN, glyco-diosgenin.

**Figure S5:**
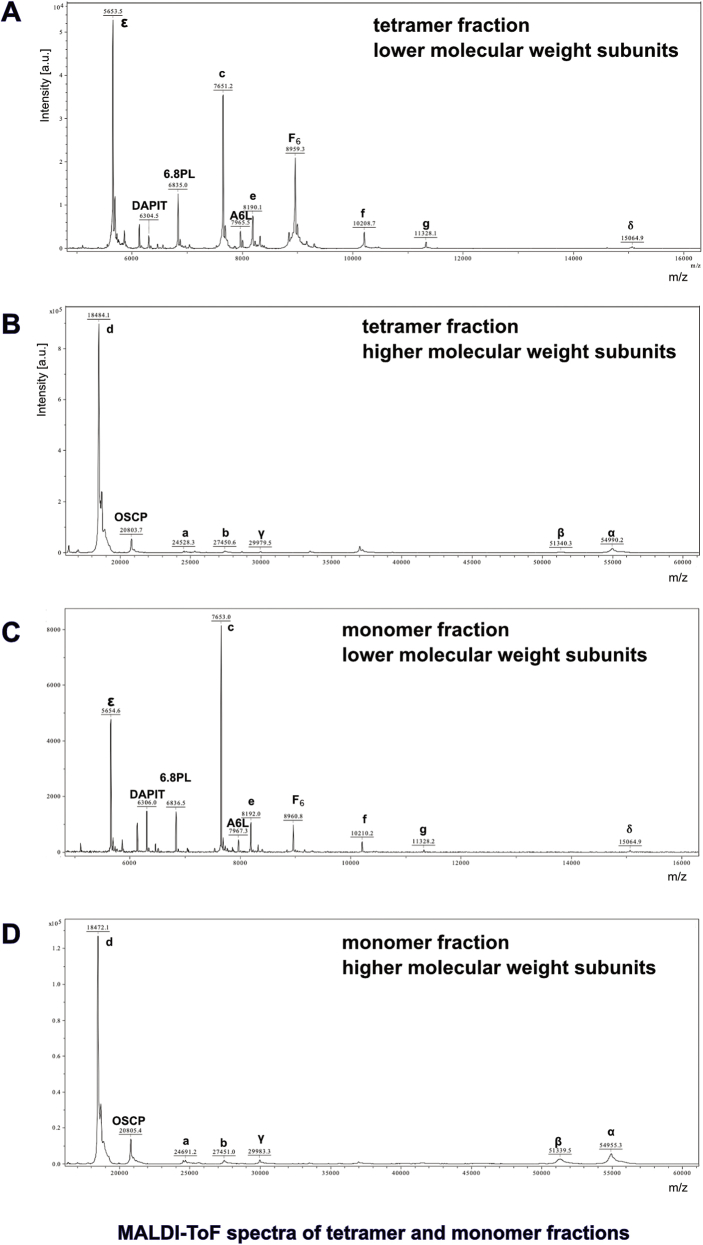


A third, continuous sucrose density gradient ultracentrifugation yielded fractions enriched in tetrameric F-ATP synthase, if run in the presence of GDN. Analysis by SDS-PAGE, CN-PAGE, and MALDI-TOF of these fractions indicate high purity and stability (Figs. 4, *A* and *B*, 6, and S5, *A* and *B*). As judged by negative stain EM the proportion of tetrameric F-ATP synthase could be sufficient for structural analysis by single particle cryo-EM (Fig. 4*C*). Immunoblotting also indicated the presence of IF1 also in the intact complexes (Fig. S6).

**Figure 5:**
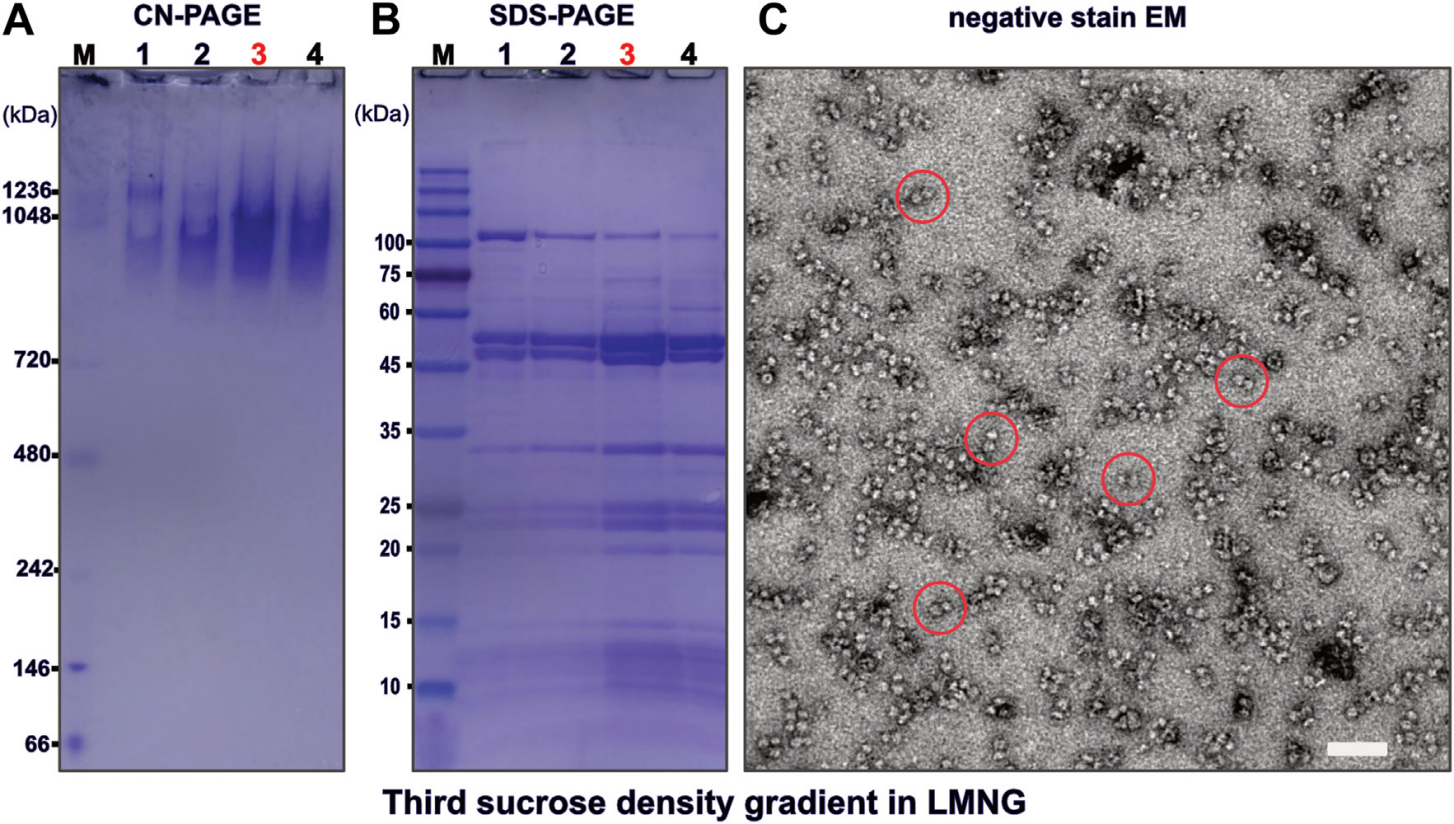
**Analysis of the third continuous sucrose LMNG density gradient fractions by native gel electrophoresis, denaturing gel electrophoresis, and negative stain electron microscopy**. *A*, clear native gel electrophoresis of fractions collected from bottom to top of the gradient with the fraction enriched in monomeric F-ATP synthase and used for negative stain electron microscopy marked in *red font*. The molecular weight marker lane on the far left is marked by M. *B*, denaturing gel electrophoresis of fractions collected from bottom to top of the gradient as analyzed in (*A*). The fraction enriched in monomeric F-ATP synthase and used for negative stain electron microscopy is marked in *red font*. Molecular weight markers are indicated on the *left* side of the gel. *C*, negative stain electron microscopy of fraction 3. Some of the F-ATP synthase complexes that appear to be monomeric F-ATP synthase are encircled in *red*. The scale bar represents 50 nm. LMNG, lauryl maltose neopentyl glycol.

**Figure 6:**
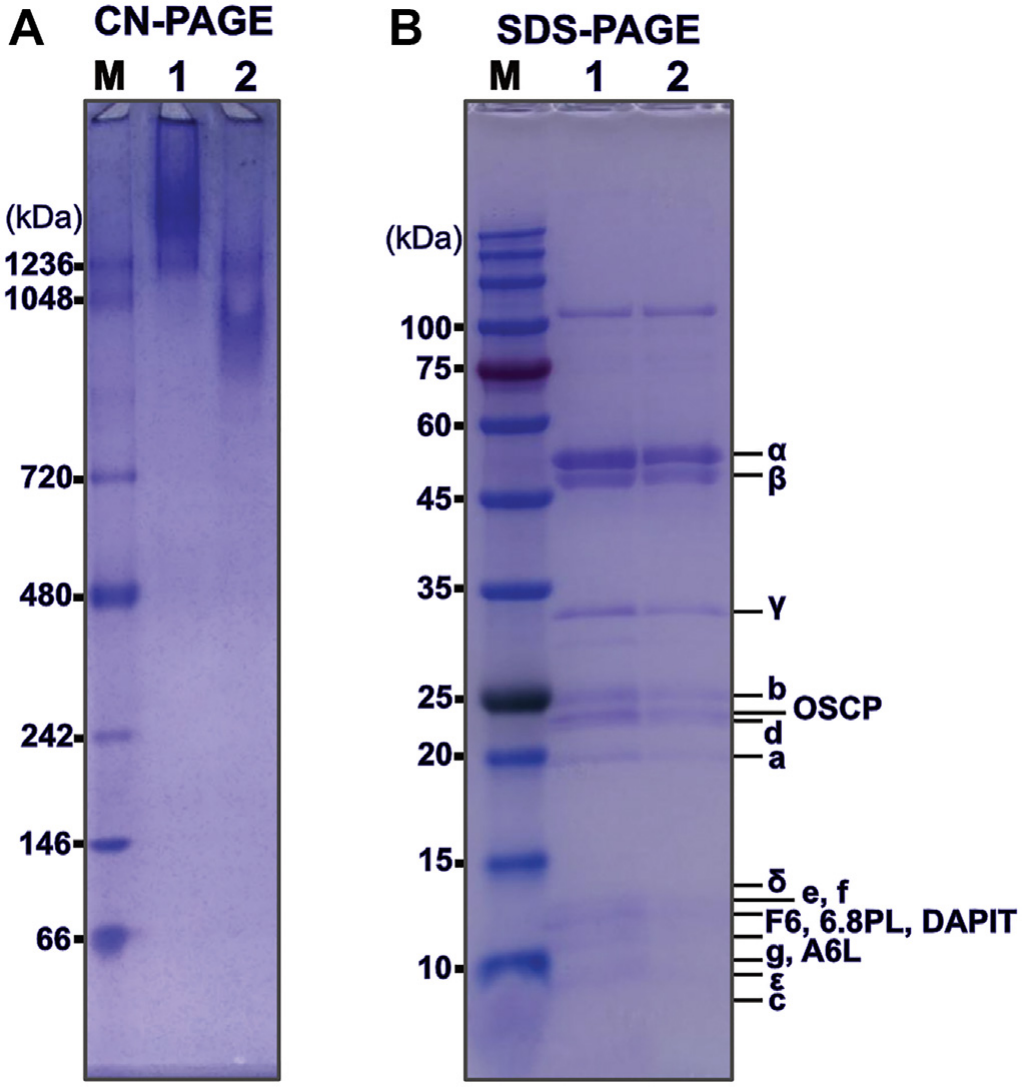
**Analysis of the final purified tetramer and monomer F-ATP synthase by native gel electrophoresis and denaturing gel electrophoresis after storage for 10 days**. *A*, clear native gel electrophoresis of the final tetramer preparation in Lane 1 and final monomer preparation in Lane 2. The molecular weight marker lane on the far left is marked by “M”. *B*, denaturing gel electrophoresis of the final tetramer preparation in Lane 1 and final monomer preparation in Lane 2. The molecular weight marker lane on the far left is marked by “M”.

A typical yield for the “tetramer” fraction is about 10 mg. When supplying LMNG to a final concentration of 0.5% to the F-ATP synthase fractions before running a third, continuous sucrose density gradient in the presence of 0.02% LMNG it was possible to obtain fractions containing almost only monomeric F-ATP synthase. Analysis by SDS-PAGE, CN-PAGE, and MALDI-TOF of these fractions indicate high purity and stability (Figs. 5, *A* and *B*, 6, and S5, *C* and *D*). Immunoblotting indicated the presence of IF1 also in the intact complexes (Fig. S6). As judged by negative stain EM the level of purity, stability, and monodispersity of the obtained monomeric F-ATP synthase should be sufficient for structural and functional studies (Fig. 5*C*). A typical yield for the “monomer” fraction is about 40 mg. Bovine F-ATP synthase stabilized in LMNG is suitable for the rapid formation of proteoliposomes by autoinsertion (40)⁠. Therefore, we expect that in this way purified monomeric F-ATP synthase to be useful for functional and structural *in vitro* studies of bovine F-ATP synthase in the context of a proton gradient.

**Figure S7:**
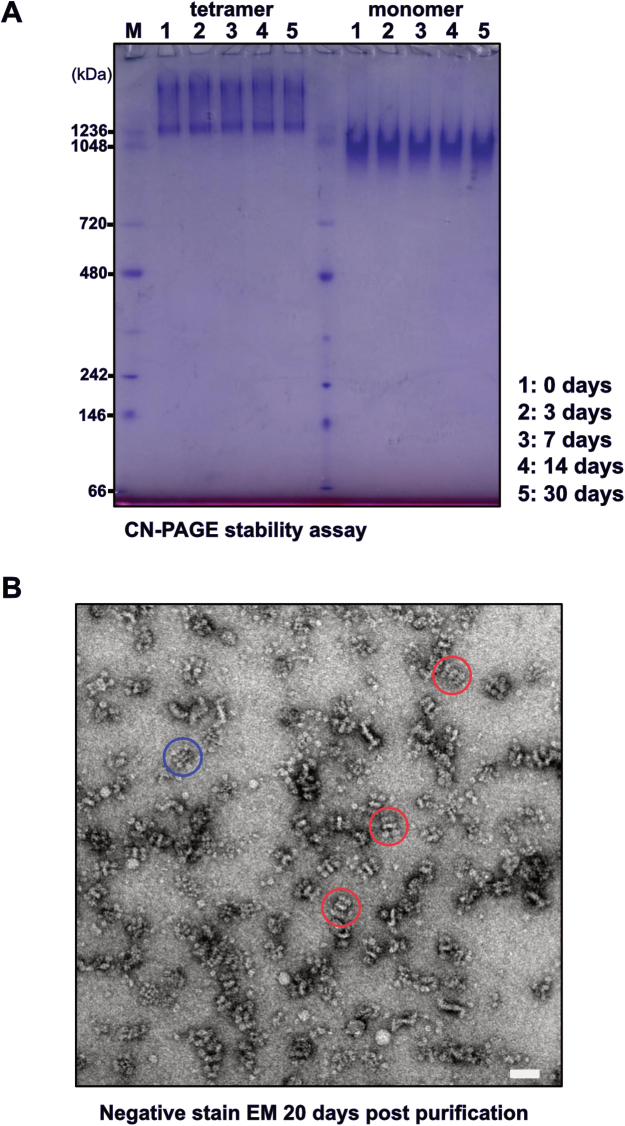


Both monomeric and tetrameric F-ATP synthase preparations exhibited long-term stability for up to a month at 4 °C and also if thawed from snap frozen aliquots (Fig. S7). A persistent contaminant appearing as a ∼100 kDa band in SDS-PAGE analysis for both tetramer and monomer preparations was analyzed by LC-MS and found to be stemming from 2-oxoglutarate dehydrogenase (Data S1). Indeed, in negative stain micrographs of the tetramer fractions particles with a shape similar to that of 2-oxoglutarate dehydrogenase can be observed (Figs. 4*C* and S7*B*). Both “tetramer” and “monomer” fractions can be concentrated and buffer exchanged by PEG precipitation for which PEG 20,000 was found to be most suitable among all PEGs tested.

## Discussion

Protein purification aims to isolate the target complex from its surrounding cellular milieu to allow its *in vitro* investigation in the absence of interference from other cellular components. As a result, experimental data can be obtained that are sufficiently interpretable to expand our biological understanding of the target complex. For a successful implementation of this reductionist approach to molecular biology, ideally, the target complex is purified from its cellular environment to homogeneity without compromising its structural and functional integrity. In the case of mammalian F-ATP synthase, this is a formidable task as a consequence of its complex and fragile multisubunit composition, which is known to be very sensitive to the disruptive effect of conventional detergents and also affinity resin, presumably due to the loss of bound lipids (29, 51)⁠. A further complication stems from the circumstance that F-ATP synthase is a rotary motor nanomachine that is sensitive to mechanical stress. Moreover, the dynamic oligomeric states of mammalian F-ATP synthase—ranging from higher oligomers to tetramers, dimers, and monomers (52)—render the purification of oligomeric pure states challenging. However, conclusive *in vitro* experiments that are able to answer important questions on higher functions mediated by the supernumerary subunits and the oligomeric state itself clearly necessitate purification procedures that preserve functional and structural integrity for all oligomeric states of interest. Traditionally the enrichment of an enzyme during purification is monitored by increasing its specific activity. Since we aimed at isolating of the IF1 bound oligomer, the opposite had to be achieved: minimized enzymatic activity with maximized band intensity in denaturing and native electrophoresis. Here, we have shown that under buffer conditions that favor IF1 binding and oligomer stabilization it is possible to obtain large amounts of pure and stable F-ATP synthase from bovine heart mitochondria without the use of chromatography steps or affinity resins. The key for the here described purification strategy is the separation of very large, higher oligomers of IF1 bound F-ATP synthase from other components of the respiratory chain after detergent-mediated solubilization of the inner mitochondrial membrane. A second sucrose step density gradient is then sufficient to obtain very pure, however, oligomeric state mixed F-ATP synthase. We demonstrate that the enrichment of two oligomeric states, namely tetramer and monomer, is feasible when using a third continuous sucrose density gradient. Since the tetrameric form of F-ATP synthase represents the smallest higher oligomer, a dimer of dimers, this opens the way to conduct *in vitro* experiments probing the functional meaning of oligomerization for the mammalian complex. Considering that questions regarding how the oligomeric state is affecting the F-ATP synthase core function of ATP synthesis remain unanswered (18), this is of interest. Furthermore, open questions concerning monomeric F-ATP synthase's ability to elicit conductance states of the mPTP might be more accessible to investigation when making use of the here reported isolation procedures. The here-described approach of isolating monomeric F-ATP synthase is unique in that the immediate input to the monomer gradient is IF1 bound oligomeric F-ATP synthase. As a consequence, the presence of mixed levels of structural integrity is avoided and a high level of structural homogeneity is provided. Another point of interest is whether oligomerization of mitochondrial F-ATP synthase requires a membrane or not (53)⁠. Oligomer contacts might involve complex bound lipids that are possibly stripped off during chromatography steps, potentially limiting the ability to reconstitute oligomeric F-ATP synthase in solution. If the presence of a membrane is not essential, we expect that in solution oligomerization might be observable for column-free purified mitochondrial F-ATP synthase. Our current structural understanding of mammalian F-ATP synthase is based on reported single particle cryo-EM structures of the porcine tetramer (11)⁠, a monomer map obtained from the bovine dimer (10)⁠, the ovine monomer (9)⁠ and the human monomer (37)⁠. Although important achievements, resolution and map resolvability are still wanting in all structures. If these limitations are at least partly the result of compositional heterogeneity, the here described column-free purification approach might be useful to improve resolution, map quality, and atomic models for both tetrameric and monomeric F-ATP synthase isolated from mammalian heart muscle tissue or human cell cultures.

An obvious drawback of our purification approach is the large amount of muscle tissue needed. Though the total amount of 600 g muscle tissue for one purification is relatively modest, study of human and mutant F-ATP synthase from cell cultures will require the adjustment of the current protocol to smaller amounts of starting material. Tetrameric IF1 bound F-ATP synthase as reported for the porcine heart muscle mitochondria and enriched in our GDN based third continuous sucrose density gradient, likely forms before the isolation of mitochondria from heart muscle tissue and not during the purification procedure. Thus we believe that tetrameric IF1 bound F-ATP synthase represents a structural entity that is also present in the living organism. We succeeded in enriching tetrameric F-ATP synthase to a level that appears to be sufficient for structure determination by single particle cryo-EM and which compares favorably for the degree of enrichment reported for the porcine and ovine heart F-ATP synthase (9, 11). However, for both structural and also functional studies enrichment to 100% is desirable and should be the ultimate aim of further improvements of the current protocol. In addition, 2-oxoglutarate dehydrogenase turned out to be a surprisingly persistent contaminant. If this stubborn presence in lieu of using three consecutive sucrose density ultracentrifugation steps is at least partially due to an unknown specific interaction with F-ATP synthase remains to be investigated. The purification procedure described here relies on the balanced use of the two novel detergents, GDN and LMNG. Their relative amount can be used to shift the oligomeric state in solution without affecting the integrity of the monomeric complex. Further variants of these two, lipid-like detergents (54) might ease the in solution manipulation of various oligomeric states and perhaps even allow for the efficient autoinsertion of higher oligomers, which unfortunately is not supported by the presence of GDN as the stabilizing detergent (40)⁠.

In recent years F-ATP synthase has attracted attention as a therapeutic drug target (55, 56)⁠. Indeed, the development of (+)-Epicatechin as a small molecule mimic of IF1 demonstrates that human F-ATP synthase could be a crucial molecular player for drug development aimed at mitochondrial disorders (19)⁠. As such, our newly developed large-scale column-free purification might evolve into a cornerstone for mitochondrial F-ATP synthase–targeted drug screening, especially, if screens are aimed at finding pharmacological compounds that target oligomeric F-ATP synthase. Our purification strategy reported here will hopefully be explored, implemented, and improved by a wide range of investigators interested in the biology of mammalian F-ATP synthase and thus contribute to our understanding of its structure and function.

## Experimental procedures

### Isolation of inner mitochondrial membrane

The procedure for the isolation of mitochondrial inner membrane used here is essentially identical to the one used for 3D crystallization of bovine cytochrome *c* oxidase as reported previously (30, 47)⁠. Slight modifications, however, were made to buffer composition and pH to stabilize IF1 binding. In brief, a single bovine heart was obtained from a local abattoir immediately after animal sacrifice. Fat tissue was removed and 600 g of lean, red meat minced using a commercial meat mincer. The mince is placed into 2.3 L of ice-cold distilled water supplied with 350 ml of ice-cold phosphate buffer (0.2 M NaP_i_, pH 7.4), and a spate of PMSF. This mixture is homogenized in a Polytron PT3100D homogenizer at 11,000 rpm for 10 min. Larger cell debris is removed by spin-down at 2800 rpm for 20 min at 4 °C with a large-scale refrigerated centrifuge (Hitachi Himac CR20G) using a R2A rotor. Subsequently, the supernatant is carefully separated from the soft pellet by straining through two sheets of gauze. Thereafter, mitochondria are pelleted from the strained supernatant by centrifugation at 8000 rpm for 25 min at 4 °C with a large-scale refrigerated centrifuge (Hitachi Himac CR20G) using a R12A F rotor and the supernatant is discarded. The mitochondrial precipitate is suspended in a buffer of 40 mM Hepes-NaOH (pH 7.3), 5 mM MgCl_2_, 5 mM DTT, 5 mM EGTA, and 0.5 mM ADP and homogenized using a loosely fitting Dounce glass homogenizer by ∼12 up-down movements. From the resulting homogenate, the inner mitochondrial membrane fraction is pelleted by ultracentrifugation for 30 min at 100,000*g* using a Hitachi Himac CP80WX ultracentrifuge with a P45A T-angle rotor. After discarding the resulting supernatant by aspiration and careful wiping of residual oil from the ultracentrifuge tubes, the total amount of inner mitochondrial membrane is weighted for later adjustment of the membrane-to-detergent ratio.

### Solubilization of inner mitochondrial membranes

Pellets of inner mitochondrial membrane are suspended in a buffer of 40 mM Hepes-NaOH (pH 7.3), 5 mM MgCl_2_, 5 mM DTT, 5 mM EGTA, and 0.5 mM ADP at a volume to weight ratio of 1 L buffer to 560 g membranes. The suspension is homogenized by approximately 12 up-down movements in a tightly fitting glass Dounce homogenizer to obtain inside-out vesicles. Note that the resulting pH of the homogenate will be slightly acidic. Solubilization of the inside-out membrane fraction is performed under constant magnetic stirrer mixing at ice-cold temperature. First, sodium deoxycholate is added to a final concentration of 0.73% (w/v) from an 11% stock solution, second, DM is added to a final concentration of 0.4% (w/v) from a 20% stock solution, after which solid KCl is added to a final concentration of 72 g/l. Finally, when the KCl salt grains are completely dissolved, GDN is added to a final concentration of 0.1% (w/v). At this point, the solution should have a pH of around 6.0. To remove insolubilized membranes the solution is ultracentrifuged for 40 min at 176,000g using a Hitachi P45A T-angle rotor. The resulting supernatant is strained through four layers of gauze and kept for equilibrium ultracentrifugation.

### Sucrose gradient equilibrium ultracentrifugation

A two-step sucrose gradient is prepared in large fixed angle rotor ultracentrifuge tubes using 16 ml of 2.3 M sucrose and 24 ml 1.6 M sucrose solubilized in a buffer of 40 mM Hepes-NaOH (pH 7.3), 5 mM MgCl_2_, 5 mM DTT, 5 mM EGTA, 0.5 mM ADP, 100 mM KCl, 0.1% DM (w/v), 0.02% GDN, and 0.02% LMNG. The solubilized inside-out vesicle solution is gently layered onto the two-step sucrose gradient and ultracentrifuged to equilibrium at 176,000*g* for 42 h at 4 °C using a Hitachi P45A T-angle rotor. The resulting gradient is collected in 2 ml fractions from the bottom of each ultracentrifuge tube using a peristaltic pump and fractions are examined for enrichment of F-ATP synthase with low ATP hydrolysis activity using a combination of SDS-PAGE, CN-PAGE, and the Pullman ATP hydrolysis activity assay (23). Fractions containing a high concentration of F-ATP synthase, but exhibiting relatively low ATP hydrolysis activity are pooled and diluted in a buffer of 40 mM Hepes-NaOH (pH 7.3), 5 mM MgCl_2_, 5 mM DTT, 5 mM EGTA, 0.5 mM ADP, 100 mM KCl, and 0.02% GDN to a final sucrose concentration that is slightly below 0.6 M sucrose as monitored using a PAL-1 pocket refractometer (Atago).

### Sucrose step gradient ultracentrifugation

A step gradient of sucrose solution is layered into large ultracentrifuge tubes from bottom-to-top using 10 ml of 1.6 M, 10 ml of 1.0 and 10 ml of 0.6 M sucrose solution with all solution prepared using a buffer of 40 mM Hepes-NaOH (pH 7.3), 5 mM MgCl_2_, 5 mM DTT, 5 mM EGTA, 0.5 mM ADP, 80 mM KCl, and 0.02% GDN. The pooled and diluted F-ATP synthase fractions are gently layered onto the step gradient and ultracentrifuged at 176,000*g* for 20 h at 4 °C using a Hitachi P45A T-angle rotor. The resulting gradient is collected in 2 ml fractions from the bottom of each ultracentrifuge tube using a peristaltic pump, and fractions are examined for enrichment of F-ATP synthase by SDS-PAGE and CN-PAGE. Fractions enriched in F-ATP synthase are pooled and diluted to 19% sucrose as monitored using a PAL-1 pocket refractometer (Atago) in a buffer of 40 mM Hepes-NaOH (pH 7.3), 5 mM MgCl_2_, 5 mM DTT, 5 mM EGTA, 0.5 mM ADP, 100 mM KCl, and 0.02% GDN.

### Continuous gradient ultracentrifugation for F-ATP synthase tetramer enrichment

Using a Gradient Master 108, Biocomp a continuous 40 to 20% sucrose gradient is prepared in swing-out rotor tubes (Open-Top Thinwall Ultra-Clear Tube, 25 × 89 mm) with a buffer composition of 40 mM Hepes-NaOH (pH 7.3), 5 mM MgCl_2_, 5 mM DTT, 5 mM EGTA, 0.5 mM ADP, 100 mM KCl, and 0.02% GDN. The diluted F-ATP synthase fractions are gently layered onto the gradient and ultracentrifuged at 113,000*g* for 20 h using a swing rotor P28S (Hitachi). The resulting gradient is collected in 2 ml fractions from the bottom of each ultracentrifuge tube using a peristaltic pump and fractions are examined for enrichment of tetrameric F-ATP synthase by SDS-PAGE, CN-PAGE, and negative stain EM.

### Continuous gradient ultracentrifugation for F-ATP synthase monomer enrichment

Using a Gradient Master 108, Biocomp a continuous 40 to 20% sucrose gradient is prepared in swing-out rotor tubes (Open-Top Thinwall Ultra-Clear Tube, 25 × 89 mm) with a buffer composition of 40 mM Hepes-NaOH (pH 7.3), 5 mM MgCl_2_, 5 mM DTT, 5 mM EGTA, 0.5 mM ADP, 100 mM KCl, and 0.02% LMNG. For deoligomerization, the pooled and diluted F-ATP synthase fractions are supplemented with LMNG to a final concentration of 0.5% (w/v) and incubated for 2 h. Subsequently, the solution is gently layered onto the gradient and ultracentrifuged at 113,000*g* for 20 h using a swing-out rotor P28S (Hitachi). The resulting gradient is collected in 2 ml fractions from the bottom of each ultracentrifuge tube using a peristaltic pump and fractions are examined for enrichment of monomeric F-ATP synthase by SDS-PAGE, CN-PAGE, and negative stain EM.

### Concentration and sucrose removal by PEG precipitation

Pooled fractions enriched in tetrameric or monomeric F-ATP synthase are supplemented dropwise with PEG 20,000 at room temperature to a final concentration of 8% and mixed by inversion until it turned cloudy. Subsequently, the mixture is spun down at 15,000 rpm for 20 min at 4 °C (TOMY M X-307). The resulting supernatant is discarded and the remaining pellet dissolved in 30 μl of 40 mM Hepes-NaOH (pH 7.3), 5 mM MgCl_2_, 5 mM DTT, 5 mM EGTA, 0.5 mM ADP, 100 mM KCl, and 0.02% GDN or 0.02% LMNG while mixing gently by finger tapping until being completely dissolved.

### Negative stain EM

An aliquot of 3.0 μl of enriched fraction was diluted x100 and applied to glow-discharged (5 mA, 10 s, Eiko LifeSciences) continuous carbon film coated copper grids (Nisshin EM). Staining was performed by applying 3.0 μl 2% uranyl acetate solution. After incubation for 30 s, staining solution was blotted using filter paper (Whatman #1; Whatman) and air dried. Specimen was inspected using a H-7650 HITACHI transmission electron microscope at 80 kV acceleration voltage equipped with a 1 × 1K Tietz FastScan- F114 CCD camera.

### Denaturing SDS gel electrophoresis

SDS-PAGE was performed using 10 to 20% continuous e-PAGEL HR (ATTO Technology). For each fraction, 5 μl was applied and electrophoresis conducted using a denaturing electrophoresis buffer (3.0 g Tris, 14.4 g glycine and 1.0 g SDS in 1 L Milli-Q water). Protein bands were visualized by SimplyBlue SafeStain (Invitrogen). The EzProtein Ladder (Atto) was used as a molecular weight marker.

### Clear native gel electrophoresis

Typically, 4 μl of sample was applied to gel pockets and native gel electrophoresis performed at 4 °C using 3 to 12% Bis-Tris gels from Invitrogen and native running buffers from SERVA running at 150 CV for 90 min (57). As a molecular weight marker, NativeMark Unstained Protein Standard (Invitrogen) was used in all clear native gel electrophoresis experiments. Gels were stained by SERVA Blue R staining kit (Serva).

### Pullman ATP hydrolysis activity assay

To monitor ATP hydrolysis activity of collected gradient fractions an ATP-regenerating enzyme-coupled assay was used (23)⁠. The ATPase activity of purified F-ATP synthase was assessed at room temperature using a NADH-coupled assay. The assay mixtures contained 40 mM K・Pi (pH 7.8), 150 mM KCl, 2 mM MgCl_2_, 2.5 mM phosphoenolpyruvate, 100 μg/ml pyruvate kinase, 100 μg/ml lactate dehydrogenase, and 0.2 mM NADH, and a range of concentrations of ATP・Mg^2+^. The reaction was initiated by adding 0.01 mg of F-ATP synthase fraction to 2 ml of the reaction mixture. The hydrolysis of ATP by the F-ATP synthase was followed by NADH oxidation at 340 nm at 20 °C in the absence or presence of oligomycin.

### Determination of protein concentration

Protein concentration was measured using a NanoDrop Lite (Thermo Fisher Scientific) by UV absorbance at 280 nm.

### Detection of IF1 by Western blot

Proteins were transferred to methanol-activated polyvinylidene fluoride membranes (Immobilon-P, Millipore) in xCell SureLock Mini-Cells under 9 V constant voltage for 30 min at room temperature. Blots were blocked with 5% skim milk in TBS-T (1 ml/l Tween-20/TBS) for 1 h at 4 °C and subsequently washed three times at 4 °C for 10 min with PBS-T. Incubation with primary antibody Anti-ATPase Inhibitory Factor1/IF1 anti body[5E2D7] (Abcom) diluted 1000× was done for 1 h at room temperature. After washing three times with TBS-T for 10 min membranes were probed using the secondary antibody Anti-mouse immunoglobulin G Horseradish Peroxidase Linked Whole Antibody (from sheep, GE HealthCare) at 1000× dilution for 1 h at room temperature. Detection was achieved using Chemi-Lumi One super (Nacalai Tesque) and the ImageQuant LAS500 (GE HealthCare) detector.

### Subunit detection by MALDI-TOF

Final fractions of both tetramer and monomer purification were analyzed using MALDI-TOF mass spectrometry (MS) with a Bruker rapifleX MALDI-TOF system (Bruker Daltonics) as previously described (58). A sinapinic acid (trans-3,5-dimethoxy-4-hydroxycinnamic acid, Bruker Daltonics) saturated solution in 33% acetonitrile/67% water containing 0.1% *trifluoroacetic acid* was prepared. A 3-hydroxypicolinic acid (Bruker Daltonics) saturated solution was also prepared. A matrix solution was prepared with 3:1 volumes of each solution. A 0.5 ml volume of each sample was mixed with 0.5 ml of the matrix solution and air-dried at room temperature on the MALDI plate for analysis.

### Identification of 100 kDa contaminant by LC-mass spectrometry

The contaminant 100 kDa protein band was excised from an SDS-PAGE gel, reduced with DTT, and alkylated with acrylamide for analysis by LC-MS as described previously (59). The protein was digested in gel with trypsin (tosyl phenylalanyl chloromethyl ketone treated; Worthington Biochemical) at 37 °C overnight. An aliquot of the digestion mixture was separated using a nano-ESI spray column (NTCC-360, 0.075 mm internal diameter × 105 mm length, 3 μm, Nikkyo Technos Co) at a flow rate of 300 nl/min and applied online to a Q-Exactive Mass Spectrometer (Thermo Fisher Scientific) with a nanospray ion source. MS and MS/MS data were acquired using the data-dependent Top 10 method. The obtained MS/MS data were searched against the NCBInr 20160711 database with Mascot Version 2.5 software (Matrix Science; https://www.matrixscience.com/mascot_support_v2_5.html) using the following parameters: taxonomy, mammalian; type of search, MS/MS ion; enzyme, trypsin; fixed modification, none; variable modifications, acetyl (protein N-term), deamidated (NQ), Gln- >pyro-Glu (N-term Q), oxidation (M), propionamide (C); mass values, monoisotopic; peptide mass tolerance, ± 15 ppm; fragment mass tolerance, ± 20 mmu; max missed cleavages, 3; instrument type, electrospray ionisation-TRAP. Proteome Discoverer 2.2 (https://www.thermofisher.com/jp/ja/home/industrial/mass-spectrometry/liquid-chromatography-mass-spectrometry-lc-ms/lc-ms-software/multi-omics-data-analysis/proteome-discoverer-software.html) was used to generate peak list and the expectation value for accepting individual spectra in Mascot search was < 0.05.

## Data availability

All data is available upon reasonable request to the corresponding authors.

## Supporting information

The article contains supporting information.

## Conflict of interest

A patent application has been submitted by C. J., Y. M., C. G. and Kyoto University based on the results of this study.

## Figures and Tables

**Figure S6:**
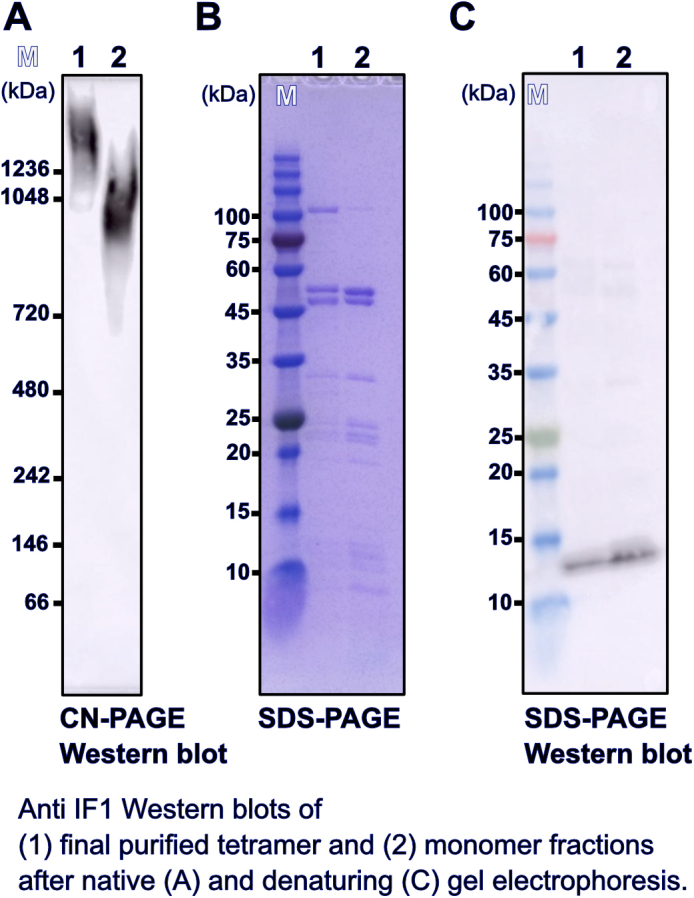


**Figure S6 Data:**
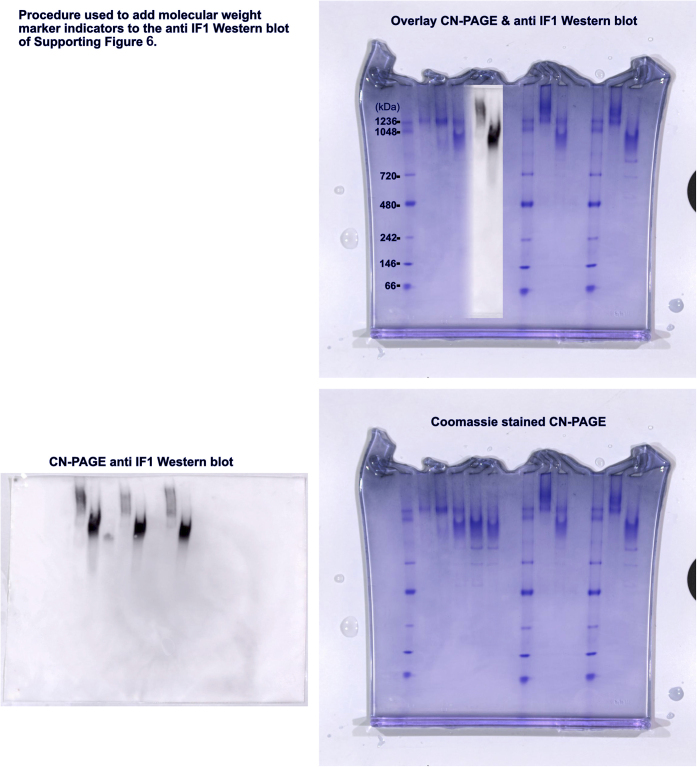


## Supporting information

SI_data_1

Supplemental_Figure_Table_Captions
