# Supplementary material for: Large-scale column-free purification of bovine F-ATP synthase
Source: J Biol Chem. 2023 Dec 28;300(2):105603. doi: 10.1016/j.jbc.2023.105603 (PMC10851226; doi:10.1016/j.jbc.2023.105603)
Supplement: Supplemental_Figure_Table_Captions [file mmc2.docx]

**Supplemental Figure Table Captions**

**Supplemental Figure 1: Gallery of experimental steps used to go from bovine heart muscle tissue to colorless F-ATP synthase oligomer enriched sucrose density gradient fractions.** A) Typical bovine heart used for F-ATP synthase purification. B) Muscle cell crushing by Polytron mixing. C) Mitochondrial inner membrane pelleting by ultracentrifugation. The membrane pellet is indicated by a blue arrow on the lower left in 'after' and residual oil can be seen as a white-yellow substance on the upper right in 'after'. D) Detergent driven mitochondrial inner membrane solubilization during stirring on ice. E) Pelleting of non-solubilized material by ultracentrifugation. F) The first sucrose density gradient ultracentrifugation by equilibrium centrifugation for the separation of IF1 bound higher oligomer F-ATP synthases from other components of the respiratory chain. Left side: after loading; right side: after ultracentrifugation. G) The second density gradient ultracentrifugation using a step gradient for further removal of contaminants. Left side: after loading; right side: after ultracentrifugation.

**Supplemental Figure 2: Western blot against F-ATP synthase β subunit of the first gradient fractions.** Fractions used are identical to those of Figure 2. The β subunit is clearly detected in all fractions of the gradient including the lower, almost colorless fractions. Fraction number 6, which was used for further purification is marked in red font.

**Supplemental Figure 3: ATP hydrolase activity of fractions collected after the first sucrose density ultracentrifugation.** Fractions 5 and 6 exhibit very low ATPase activity indicating the presence of IF1 bound F-ATP synthase.

**Supplemental Figure 4: Western blot against IF1 of the first gradient fractions.** Fractions used are not identical but analogous to those of Figure 2 as the fractions analyzed here are from a different purification lot. Fraction 8 here would be analogous to fraction 6 in Figure 2. IF1 is clearly detected in the fractions of the gradient that contain a high concentration of F-ATP synthase. Fraction number 8, which was used for further purification is marked in red font.

**Supplemental Figure 5: Detection of all expected subunits of F-ATP synthase in the final tetramer and monomer fractions by MALDI-ToF.**

MALDI-ToF spectra of lower and higher molecular weight of the tetramer fractions (A) and (B) respectively. MALDI-ToF spectra of lower and higher molecular weight of the monomer fractions (C) and (D) respectively.

**Supplemental Figure 6: Western blot against IF1 of the final fractions.**

Immunoblotting against IF1 using final purified tetramer (1) and monomer (2) fractions. After clear native PAGE (A) and after denaturing SDS-PAGE (C). The molecular weight marker indicator was added to (A) by overlaying an image of the immunoblotted gel with that of a separate gel run under the same conditions. See also supplementary data 2.

**Supplemental Figure 7: Long term stability test by CN-PAGE and negative stain EM.**

(A) CN-PAGE of both tetramer and monomer fractions did not indicate any change of sample size over the course of storage at 4 degrees Celsius for 30 days. (B) Negative stain EM of the tetramer fraction after 20 days storage at 4 degrees Celsius detects a large number of tetrameric F-ATP synthase complexes of which three are encircled in red. A putative 2-oxoglutarate dehydrogenase complex is encircled in blue. Scale bar 50 nm.

**Supplemental Table 1**

All specific activities were measured in triples of technical repeats using the same purification batch. Note the limited meaning of hydrolysis activity measurements for F-ATP synthase complexes with tightly bound IF1.
