# Supplementary material for: Large-scale column-free purification of bovine F-ATP synthase
Source: J Biol Chem. 2023 Dec 28;300(2):105603. doi: 10.1016/j.jbc.2023.105603 (PMC10851226; doi:10.1016/j.jbc.2023.105603)
Supplement: Supplemental Table 1 [file mmc3.docx]

**Supplemental Table 1**

| **Fractions of**  **1st sucrose**  **gradient** | **specific activity**  **(μmol・min^-1^・mg^-1^)** | **final tetramer**  **fraction** | **specific activity**  **(μmol・min^-1^・mg^-1^)** | **final monomer**  **fraction** | **specific activity**  **(μmol・min^-1^・mg^-1^)** |
| --- | --- | --- | --- | --- | --- |
| 5 | 0.088±0.013 | - oligomycin | 0.856±0.215 | - oligomycin | 0.260±0.030 |
| 6 | 0.124±0.028 | + oligomycin | 0.448±0.018 | + oligomycin | 0.089±0.089 |
| 7 | 0.186±0.030 | pH 8.4 | 0.956±0.08 | pH 8.4 | 1.11±0.06 |
| 8 | 0.084±0.010 |  |  |  |  |
| 9 | 0.065±0.012 |  |  |  |  |
| 10 | 0.054±0.002 |  |  |  |  |

All specific activities were measured in triples of technical repeats using the same purification batch. Note the limited meaning of hydrolysis activity measurements for F-ATP synthase complexes with tightly bound IF1.
